# Low Cytochrome Oxidase 1 Links Mitochondrial Dysfunction to Atherosclerosis in Mice and Pigs

**DOI:** 10.1371/journal.pone.0170307

**Published:** 2017-01-25

**Authors:** Paul Holvoet, Maarten Vanhaverbeke, Benjamine Geeraert, Dieuwke De Keyzer, Maarten Hulsmans, Stefan Janssens

**Affiliations:** 1 Department of Cardiovascular Sciences, Atherosclerosis and Metabolism Unit, KU Leuven, Belgium; 2 Department of Clinical Cardiology, Leuven, Belgium; Brigham and Women's Hospital, Harvard Medical School, UNITED STATES

## Abstract

**Background:**

Cytochrome oxidase IV complex regulates energy production in mitochondria. Therefore, we determined the relation of COX genes with atherosclerosis in mice and pigs.

**Methods and results:**

First, we compared atherosclerosis in the aortic arch of age-matched (24 weeks) C57BL/6J control (n = 10), LDL-receptor deficient (n = 8), leptin-deficient ob/ob (n = 10), and double knock-out (lacking LDL-receptor and leptin) mice (n = 12). Low aortic *mitochondria-encoded cytochrome oxidase 1* in obese diabetic double knock-out mice was associated with a larger plaque area and higher propensity of M1 macrophages and oxidized LDL. Caloric restriction increased *mitochondria-encoded cytochrome oxidase 1* and reduced plaque area and oxidized LDL. This was associated with a reduction of titer of anti-oxidized LDL antibodies, a proxy of systemic oxidative stress. Low of *mitochondria-encoded cytochrome oxidase 1* was related to low expression of peroxisome proliferative activated receptors α, δ, and γ and of peroxisome proliferative activated receptor, gamma, co-activator 1 alpha reflecting mitochondrial dysfunction. Caloric restriction increased them. To investigate if there was a diabetic/obesity requirement for *mitochondria-encoded cytochrome oxidase 1* to be down-regulated, we then studied atherosclerosis in LAD of hypercholesterolemic pigs (n = 37). Pigs at the end of the study were divided in three groups based on increasing LAD plaque complexity according to Stary (Stary I: n = 12; Stary II: n = 13; Stary III: n = 12). Low *mitochondria-encoded cytochrome oxidase 1* in isolated plaque macrophages was associated with more complex coronary plaques and oxidized LDL. Nucleus-encoded cytochrome oxidase *4I1* and cytochrome oxidase *10* did not correlate with plaque complexity and oxidative stress. In mice and pigs, *MT-COI* was inversely related to insulin resistance.

**Conclusions:**

Low *MT-COI* is related to mitochondrial dysfunction, oxidative stress and atherosclerosis and plaque complexity.

## Introduction

It has been proposed that mitochondrial decline resulting in mitochondrial oxidative stress contributes to the development of age-related metabolic and cardiovascular diseases [1]. Impairment of the cytochrome *c* oxidase (COX), or complex IV, results in reactive oxygen intermediates promoting oxidative stress [2]. This bigenomic complex is composed of subunits coded by both mitochondrial and nuclear DNA. A coordinated expression of these subunits provides cells with different modes of regulation of enzyme content in mitochondria. Of the thirteen subunits of the mammalian complex IV, the mitochondrial genome encodes subunits 1, 2 and 3, which form the catalytic core of the enzyme [3]. *MT-COI* is the first gene in the polycistronic mitochondrial DNA and a single missense mutation in mouse *Mt-co1* was associated with loss of COX activity [4], despite normal assembly of the complex IV, and with increased mitochondrial oxidative stress in cells *in vitro* [5].

Recently, low expression of cytochrome oxidase IV was found to be associated with mitochondrial dysfunction in obesity and diabetes [6–8]. We found that low COX4I1 and low COX10 in monocytes and adipose tissues of patients and in adipose tissues of double-knock-out mice were associated with obesity and type 2 diabetes [9]. However, low COX4I1 and low COX10 in monocytes and monocyte-derived exosomes were not associated with risk of future cardiovascular events. In contrast, low *MT-COI* predicted future events, even adjusting for established cardiovascular risk factors and inflammation markers [10]. This association was observed independent of obesity.

Aim: We here turned to preclinical models to better understand how COX genes relate to atherosclerotic burden and plaque features in obese mice and non-obese pigs. In pigs, we measured its expression in isolated macrophages. We observed that reduced *MT-COI* was related with higher atherosclerotic plaque burden and oxidative stress and with M1 macrophages. It was also linked with decreases in the peroxisome proliferative activated receptors (PPARs) and in peroxisome proliferative activated receptor, gamma, co-activator 1 alpha (PGC-1α) reflecting mitochondrial dysfunction [11–14].

### Animal experiments

Animal experiments conformed to the Guide for the Care and Use of Laboratory Animals published by the US National Institutes of Health (NIH Publication No. 85–23, revised 1996). They were approved by the Institutional Animal Care and Research Advisory Committee of the KU Leuven (Permit Number: P087).

Homozygous LDL receptor knockout mice (LDLR^−/−^), heterozygous ob/+, and C57BL6 mice were purchased from Jackson Laboratory (Bar Harbor, Maine). LDLR^−/−^ mice were backcrossed into a C57BL6 background to the tenth generation and had 98.4% C57BL6 background. To obtain leptin deficiency (ob/ob) on a background of LDLR deficiency, LDLR^−/−^ and ob/+ mice were crossed, and the F1 progeny of this mating (LDLR^−/+^;ob/+) were then crossed to obtain mice that had either zero, one, or both normal LDLR alleles and were leptin-deficient (LDLR^−/−^;ob/ob, LDLR^+/−^;ob/ob, and LDLR^+/+^;ob/ob, respectively) as well as control LDLR^−/−^, LDLR^+/−^, and wild-type mice. We refer to LDLR^−/−^;ob/ob as double knock-out or DKO mice. All offspring were genotyped by polymerase chain reaction (PCR) techniques as previously described [15,16]. In the first mouse study, we compared age-matched (24 weeks) C57BL/6J control mice (n = 10), with LDLR^-/-^, n = 8), ob/ob (n = 10), and DKO mice (n = 12). In the second study, control DKO mice were compared with caloric restricted mice (n = 10). Food intake in the latter mice was restricted to 2.5 g/d for 12 weeks between 12 and 24 weeks of age compared to ≈5.7 g/day for control DKO mice. After an overnight fast, blood was collected by puncturing the *vena cava*. Plasma was analyzed and atherosclerosis was assessed as detailed in supplement. Mice were euthanized by a single intra-peritoneal injection of 60 mg/kg Nembutal (Abbott Laboratories, North Chicago, IL, USA) [17]. Detailed information is given in S1 File.

Miniature pigs (Charles River Laboratories, Cléons, France) were bred and maintained at KU Leuven as described previously [18,19]. Pigs (n = 37) were fed an atherogenic diet, containing 4% cholesterol, 14% beef tallow, and 2% hog bile, administered at an amount of 1 kg/d starting at a mean age of 18 weeks and continued for 12 weeks in 17, for 24 weeks in 8 and for 36 weeks in 10 pigs. Plasma was analyzed and atherosclerosis was assessed as detailed in supplement. Pigs were euthanized by injecting an overdose of propofol and saturated potassium chloride [20]. Detailed information is given in S1 File.

### RNA isolation and quantitative real-time PCR analysis

Total *RNA* was extracted from aorta or from macrophages isolated by laser capture, and first-strand cDNA was generated. qPCR using Fast SYBRGreen master mix, was performed as described previously [9,21]. Detailed information is given in S1 File.

### Western Immunoblotting

The whole mouse thoracic aorta was homogenized. Protein concentrations were determined. Proteins (16.3 μg/well) were separated by SDS-PAGE in a Criterion^TM^ Vertical Electrophoresis Cell (Bio-Rad, 1656001). Blotting was performed in a Criterion^TM^ Blotter using thick blot paper (Bio-Rad, 170–4085) and Immobilon-FL Transfer Membrane (Millipore, IPFL00010). Membrane was incubated with primary antibodies: rabbit anti-Mt-co1 (Abcam, ab203912; shows a 57 kDa instead of 30kDa protein according to Abcam) and rabbit anti-β-actin (Cell Signaling, 4970L; shows a 45 kDa protein). Signals were visualized using Odyssey CLx imaging system and analyzed with Image studio Ver 5.0.

### Statistical analysis

Two groups were compared with an unpaired Mann-Whitney test. More than two groups were compared with two-tailed Kruskal-Wallis nonparametric ANOVA followed by Dunn’s comparison of all groups (GraphPad Prism 6). P-values of less than 0.05 were considered as statistically significant.

## Results

### Mouse atherosclerosis and gene expressions in aorta

Compared with C57BL/6J control mice, double knock-out (DKO) mice, DKO mice had lowest adiponectin levels, although their weight was not different from ob/ob mice. DKO mice had highest HOMA-IR and cholesterol and triglyceride levels (Table 1). They also had the largest atherosclerotic lesions in their aortic arch mainly due to higher percentages of lesion areas containing oxidized LDL.

**Table 1:** Blood variables, atherosclerosis and gene expression in aorta of lean and obese mice.

	C57BL/6J	LDLR^-/-^	Ob/Ob	DKO	ANOVA
	N = 10	N = 8	N = 10	N = 12	
**A. Weight and blood variables**					
Gender (male, n)	5	4	5	6	
Weight (g)	27±4.4	26±5.0	68±3.5^***^^/^^†††^	63±3.3^**^^/^^†^	P<0.001
ADN (μg/mL)	5.5±1.9	4.0±1.0	4.7±0.9	2.8±1.7^**^^/^^‡^	P<0.01
Glucose (mg/dL)	76±12	77±6.8	117±24^**^^/^^††^	136±45^***^^/^^†††^	P<0.001
IPGTT ^a^	35±1.5	49±7.3	56±12^**^	84±25^***^^/^^†^	P<0.001
Insulin (mU/L)	72±15	51±17	94±60	181±76^**^^/^^†††^^/^^‡^	P<0.001
HOMA-IR	1.4±0.35	0.96±0.31	2.7±1.6	6.6±4.1^**^^/^^†††^	P<0.001
Total C (mg/dL)	54±13	155±44^**^	63±22	467±89^***^^/^^‡‡‡^	P<0.001
TG (mg/dL)	43±16	45±20	28±4.9	196±45^**^^/^^††^^/^^‡‡‡^	P<0.001
**B. Atherosclerosis**					
Plaque volume ^b^	ND	19±15	ND	87±22^†††^	- -
MQ (%)	ND	21±11	ND	28±11	- -
Oxidized LDL (%)	ND	5.0±3.0	ND	12±5.0^††^	- -
SMC (%)	ND	5.0±5.2	ND	8.2±6.2	- -
**C. Gene expressions in aorta**					
*Mt-co1*	1.05±0.36	2.63±1.23	1.07±0.25^††^	0.54±0.18^*^^/^^†††^^/^^‡‡^	P<0.001
*Tfam*	1.02±0.21	1.04±0.45	0.88±0.26	0.63±0.13^*^	P<0.001
*Cox4i1*	1.06±0.35	0.91±0.47	0.47±0.04^***^^/^^†^	0.64±0.16^*^	P<0.001
*Cox10*	1.06±0.36	1.13±0.55	0.70±0.15^*^	0.82±0.18	P<0.01
*Mcp1*-to-*Cd206* ratio	ND	1.00±0.24	ND	3.86±1.16^†††^	- -

Data shown are means ± SD. Abbreviations: ADN: adiponectin; AUC: area under curve; C: cholesterol; HOMA-IR, homeostasis model assessment of insulin resistance; IPGTT: intraperitoneal glucose tolerance test; MQ: SMC: smooth muscle cells; TG: triglyceride. ^a^ Area under curve x 10^3^; ^b^ total plaque volumes were expressed in x 10^−3^ μm³, measured on Oil Red O stained sections. ND: detectable. Because C57BL6 and ob/ob mice did not have atherosclerotic plaques; ANOVA was not performed.

^*^P < 0.05

^**^P < 0.01 and

^***^P < 0.001 compared with C57BL/6J;

^**†**^*P* < 0.05

^**††**^*P* < 0.01

^**†††**^*P* < 0.001 compared with LDLR^(-/-)^;

^‡^*P* < 0.05

^‡‡^*P* < 0.01

^‡‡‡^*P* < 0.001 compared with ob/ob.

**Fig 1:**
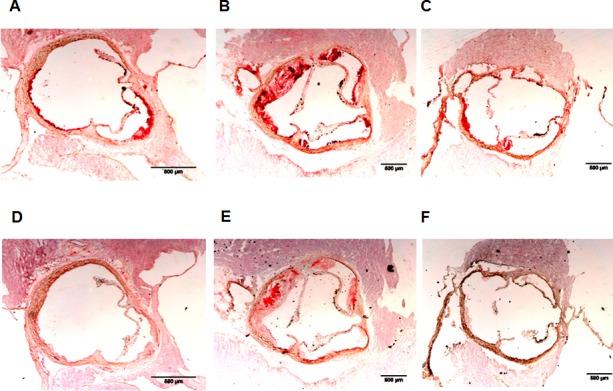
Atherosclerosis in LDLR^-/-^ and placebo DKO and caloric-restricted DKO mice. **A, B, and C:** Representative sections of atherosclerotic plaques are shown in which macrophages are stained with anti-MAC-3 antibody. D, E, and F: Representative sections of atherosclerotic plaques are shown in which oxidized LDL is stained with mAb4E6. Plaques in LDLR^**-/-**^ mice are shown in A and D, in placebo DKO mice in B and E, and in caloric restricted DKO mice in C and F.

**Fig 2:**
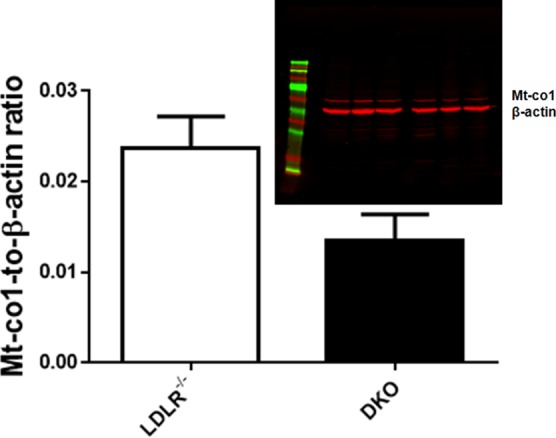
Western blot showing Mt-co1 and β-actin protein in representative extracts of aortas of LDLR^-/-^ and placebo DKO. The Mt-co1-to-β-actin ratio was higher in LDLR^-/-^ than in placebo DKO mice. **p<0.01 compared to LDLR^-/-^ mice.

**Fig 3:**
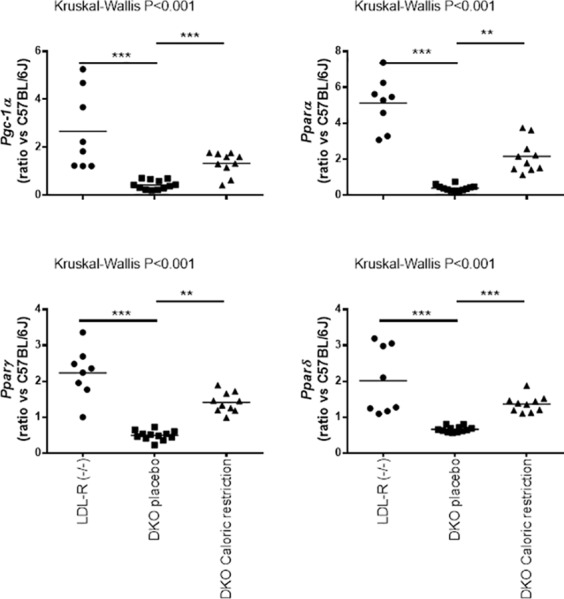
Expression of Pgc-1α and PPARs in aortic extracts of LDL-receptor deficient and placebo DKO and caloric-restricted DKO mice. Scatter plots show expression of *Pgc-1α*, *Pparα*, *Pparγ* and *Pparδ* in LDL-receptor (n = 8), placebo DKO (n = 12) and caloric-restricted DKO mice (n = 10). Gene expression data are ratios compared to expressions in aortic extracts of 10 C57BL/6J mice. **p<0.01 and ***p<0.001 compared to placebo DKO mice.

Fig 1 shows representative sections of atherosclerotic plaques in aortic arch of LDLR^-/-^ and DKO mice. Although percentages of macrophages in lesions in LDLR^-/-^ and DKO mice were similar, macrophages in lesions in DKO mice were more often of type M1, evidenced by highest Mcp1-to-Cd206 ratio, than in LDLR^-/-^ mice (Table 1). Aortic *Mt-co1* and *Tfam* expressions were the lowest in DKO mice; they were the highest in LDLR^-/-^ mice (Table 1). The Mt-co1-to-β-actin protein ratio was higher in aortic extracts of LDLR^-/-^ mice than in DKO mice (Fig 2). The ratio oxidized LDL-to-macrophages was 0.49±0.28 in DKO and 0.18±0.04 (p<0.01) in LDL-R^-/-^ mice. Aortic *Cox4i1* expressions were lower in DKO mice than in C57BL/6J control mice, but were similar to those in LDLR^-/-^ and ob/ob mice. *Cox10* expression was the lowest in ob/ob mice, but its expression in DKO mice was not lower than in other strains (Table 1). The expression of *Pgc-1α* was 2.67±1.65 in aortic extracts of LDL-receptor mice and 0.43±0.19 in extracts of DKO mice, normalized to that in extracts of C57BL/6J control mice. Expression of *Pparα* was 5.12±0.14 in LDL-receptor mice and 0.40±0.17 in DKO mice. Expression of *Pparγ* was 2.23±0.70 and 0.50±0.13, respectively. Expression of *Pparδ* was 2.01±0.94 and 0.66±0.082, respectively (Fig 3).

**Fig 4:**
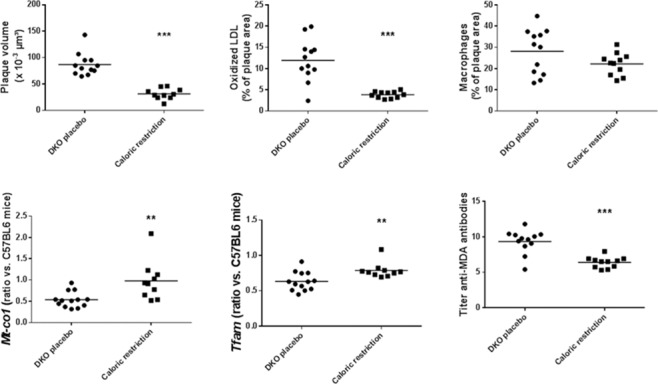
Atherosclerosis and gene expressions in placebo DKO and caloric-restricted DKO mice. Scatter plots showing plaque volume, percentage of plaque area with oxidized LDL, percentage of plaque area with macrophages, *Mt-co1* and *Tfam* RNA expression, and titer of anti-oxidized LDL antibodies, as proxy of plasma oxidative stress in placebo (n = 12) and caloric restricted (n = 10) DKO mice. Gene expression data are ratios compared to expressions in aortic extracts of 10 C57BL/6J mice. **p<0.01 and ***p<0.001 compared to placebo DKO mice.

We then investigated the effect of caloric restriction in mice. At baseline (12 weeks) characteristics of control and caloric restricted DKO mice were identical, but caloric restriction caused weight loss at 24 weeks. It also decreased insulin levels, HOMA-IR, cholesterol and triglycerides while it elevated adiponectin (Table 2). Fig 1 shows representative sections of atherosclerotic plaques in aortic arch of placebo and caloric restricted DKO mice. Under caloric restriction we also found reduced plaque volumes and percentages of oxidized LDL (Fig 4). Although percentages of macrophages were not lower, the M1-to-M2 ratio decreased from 3.86±1.16 to 0.74±0.15 (p<0.001). Also, the ratio oxidized LDL-to-macrophages decreased from 0.49±0.28 to 0.14±0.06 (p<0.001). The latter was like that in plaques in LDLR^-/-^ mice. Caloric restriction further increased *Mt-co1* and *Tfam* RNA *expression* in the aorta (Fig 4). This was associated with a decreased plasma oxidative stress, evidenced by lower titer of anti-oxidized LDL antibodies (Fig 3). Caloric restriction increased expression of *Pgc-1α* in aortic extracts of DKO mice from 0.43±0.19 to 1.33±0.48. Caloric restriction increased *Pparα* from 0.40±0.17 to 2.16±0.91, *Pparγ* from 0.50±0.13 to 1.41±0.28, and *Pparδ* from 0.66±0.082 to 1.37±0.23 (Fig 3).

**Table 2:** Effect of caloric restriction in DKO mice.

	Placebo	Caloric restriction
	N = 12	N = 10
Gender (male, n)	6	5
Weight (g)	63±3.3	35±5.0^***^
ADN (μg/mL)	2.8±1.7	5.8±1.3^**^
Glucose (mg/dL)	136±45	158±60
IPGTT^a^	84±25	84±26
Insulin (mU/L)	181±76	101±51^*^
HOMA-IR	6.6±4.1	3.1±1.9^*^
Total C (mg/dL)	467±89	393±66^*^
TG (mg/dL)	196±45	103±45^***^
Titer Abs OxLDL	9.34±1.66	6.40±0.77^***^
**B. Atherosclerosis**		
Plaque volume ^b^	87±22	31±10^***^
MQ (%)	28±11	22±5.0
OxLDL (%)	12±5.0	3.9±0.77^***^
SMC (%)	8.2±6.2	8.9±2.1

Data shown are means ± SD. Abbreviations: Abs; antibodies; ADN: adiponectin; C: cholesterol; HOMA-IR, homeostasis model assessment of insulin resistance; IPGTT: intraperitoneal glucose tolerance test; MQ: macrophages; OxLDL: oxidized LDL; SMC: smooth muscle cells; TG: triglyceride.

^*^P < 0.05

^**^P < 0.01

^***^P < 0.001 compared with control DKO

^a^ Area under curve x 10^3^

^b^ total plaque volumes were expressed in x 10^−3^ μm³, measured on Oil Red O stained sections.

*Mt-co1* (R_s_ = -0.44; p<0.01) and *Tfam* (R_s_ = -0.54; p<0.001) expressions were inversely related to titer of antibodies against oxidized LDL. *Mt-co1* (R_s_ = -0.63; p<0.001) and *Tfam* (R_s_ = -0.39; p<0.05) expressions were inversely related to *Ccl2* (*Mcp1*). *Mt-co1* but not *Tfam* expressions were inversely related to plaque oxidized LDL (R_s_ = -0.49; p<0.05). *Mt-co1* expressions were inversely related to HOMA-IR, that was positively related to plaque oxidized LDL (R_s_ = 0.69; p<0.01). *Mt-co1* (R_s_ = -0.67; p<0.001) and *Tfam* (R_s_ = -0.57; p<0.001) expressions were inversely related to triglyceride levels, which were positively related to plaque oxidized LDL (R_s_ = 0.53; p = 0.01). *Mt-co1* correlated with *Tfam* expression (R_s_ = 0.61; p<0.001).

Finally, caloric restriction increased *Cox10* (1.66±0.39 vs. 0.81±0.17; p<0.001) but not that of *Cox4i1* (0.87±0.44 vs. 0.64±0.16).

### Atherosclerosis in miniature pigs and gene expressions in isolated macrophages

**Fig 5:**
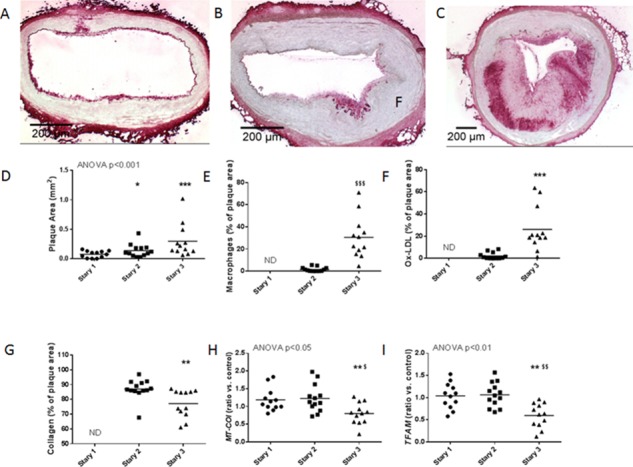
Atherosclerotic plaques in coronary arteries and gene expressions in coronary plaque macrophages in high-fat diet-fed miniature pigs. A-C: Representative sections of Stary I (A), Stary II (B) and Stary III (C) atherosclerotic plaques in coronary arteries of pigs are shown. Oxidized LDL is stained with mAb4E6. D-I: Scatter plots showing coronary plaque area (D), percentage of plaque area with M1 macrophages (E), percentage of plaque area with oxidized LDL (F), percentage of plaque area with collagen (G), and *MT-COI* (H) and *TFAM* (I) expressions in coronary artery macrophages in Stary I (n = 5), Stary II (n = 8) and Stary III (n = 10) miniature pigs. Gene expression data are ratios compared to expressions in coronary artery tissue extracts of 16 control pigs without atherosclerosis. *P<0.05, ***P<0.001 compared to Stary I; ^†^P<0.05, ^††^P<0.01 compared to Stary II.

We then investigated whether *MT-COI* was reduced in macrophages in relation to plaque complexity. We therefore studied coronary atherosclerosis in high-fat diet-fed miniature pigs, and measured gene expressions in coronary plaque macrophages isolated by laser capture. Diet pigs were categorized in 3 groups according the characteristics of their coronary atherosclerotic plaques using the Stary classification. Table 3 shows that age, gender, weight, plasma leptin, adiponectin, glucose, triglycerides, LDL-cholesterol, HDL-cholesterol, and hs-CRP were similar in 3 groups of diet-fed pigs. However, pigs with Stary III lesions had higher HOMA-IR and higher plasma levels of oxidized LDL. Fig 5 shows representative sections of Stary I, Stary II and Stary III lesions in which oxidized LDL is stained with mAb-4E6. It also shows that coronary plaque areas were not different in pigs with Stary I, or Stary II or Stary III coronary plaques. However, Stary III plaques contained more M1 macrophages, oxidized LDL, and less collagen, indicative for more unstable plaques. Interestingly, expression of *MT-CO1* and *TFAM* was lowest in macrophages isolated from Stary III coronary plaques (Fig 5). This was also associated with higher plasma oxidized LDL levels. *MT-COI* (R_s_ = -0.46; P<0.05) and *TFAM* (R_s_ = -0.53; P<0.01) were inversely related to plaque oxidized LDL. *MT-COI* (R_s_ = -0.70; P<0.001) and *TFAM* (R_s_ = -0.56; P<0.01) were inversely related to HOMA-IR. *MT-COI* (R_s_ = 0.72; P<0.001) correlated with *TFAM*.

**Table 3:** Characteristics of diet pigs according to stage of coronary atherosclerosis.

	Stary I	Stary II	Stary III	ANOVA
	N = 12	N = 13	N = 12	
**A. Characteristics**				
Age at start (weeks)	20±8	18±7	20±10	NS
Age at end (weeks)	38±18	38±6	35±6	NS
Gender (n male)	6	4	6	NS
Weight at start (kg)	23±6	22±6	24±8	NS
Weight at end (kg)	63±42	60±30	54±24	NS
Leptin (ng/mL)	12±5.1	12±13	8.2±5.6	NS
ADN (μg/mL)	10±4.4	10±4.5	10±5.2	NS
Glucose (mg/dL)	112±51	127±47	129±55	NS
Insulin (μg/L)	0.10±0.07	0.10±0.04	0.22±0.09*/^†^	<0.05
HOMA-IR	0.31±0.25	0.34±0.16	0.77±0.43^†^	<0.05
TG (mg/dL)	116±106	95±71	83±59	NS
LDL-C (mg/dL)	307±216	421±213	425±165	NS
HDL-C (mg/dL)	166±120	114±82	102±70	NS
Hs-CRP (mg/L)	2.3±2.2	1.1±0.7	1.4±0.9	NS
OxLDL (mg/dL)	0.79±0.35	1.1±0.4	1.4±0.4*	<0.05

Data shown are means ± SD. Abbreviations: ADN: adiponectin; AUC: area under curve; C: cholesterol; HOMA-IR, homeostasis model assessment of insulin resistance; OxLDL: oxidized LDL.

^*^P < 0.05 compared with control pigs

^†^P<0.01 compared with stage II.

Expression of *COX4I1* was lower in macrophages isolated from Stary III than in macrophages isolated from Stary I and Stary II plaques (0.88±0.15 vs. 1.07±0.21 and 1.45±0.40; p<0.01). However, *COX4I1* did not correlate with plaque oxidized LDL. Expression of *COX10* in macrophages was not different according to the plaque type (1.63±0.34 in Stary I, 1.39±0.20 in Stary II, and 1.12±0.28 in Stary III).

## Discussion

### Low MT-COI and atherosclerosis

In this study, we validated impaired *MT-COI* expression in 2 pre-clinical models of atherosclerosis. In obese, diabetic mice, aortic atherosclerotic lesion size inversely correlated with *Mt-co1* expression levels and was modifiable by dietary interventions. In addition, in high-fed diet pigs low *MT-COI* in macrophages was associated with coronary plaque complexity. In aggregate, our data are consistent with recent observations that macrophage mitochondrial oxidative stress promotes atherosclerosis [22] and that *MT-COI* may act as a molecular switch in this process. Indeed, novel finding of this study is that low *Mt-co1* expression was associated with a higher propensity of M1 macrophages in atherosclerotic plaques and with higher oxidative stress, both in plaques and in plasma. Whereas our mouse studies did not allow concluding if low *MT-COI* was dependent on obesity and/or diabetes, our study in miniature pigs demonstrated that low *MT-COI* was associated with plaque complexity independent of weight but dependent on insulin resistance (HOMA-IR). We previously showed that decrease in nucleus encoded *COX4I1* and *COX10* depended on obesity and insulin resistance and diabetes [22]. However, in our current study they were not related to plaque complexity and oxidative stress.

Previously, it has been shown that complex IV enzyme activity depends on the expression of genes involved in mitochondrial biogenesis [23], and that induction of the COX IV complex is part of the adaptive anti-oxidant stress response of monocytic cells to hypoxic condition [24]. For example, “small LDL” was found to be associated with impairment of the COX IV complex, high oxidative stress and impaired cholesterol efflux capacity in peripheral blood mononuclear cells *in vitro* [25]. In addition, it has been hypothesized that impairment of mitochondrial function may link oxidative stress with reduced fitness to a higher incidence of cardiovascular diseases [26]. However, the relation of individual genes in this complex with atherosclerosis and cardiovascular diseases has not been studied. Therefore, we studied this relation in two different preclinical models.

First, we compared obese diabetic mice [15]. Interestingly, plaque expression of *Mt-co1* was reduced in obese as opposed to lean mice. *Mt-co1*expressions were inversely related to HOMA-IR and triglyceride levels, and are consistent with our previous human data linking metabolic syndrome factors to high oxidative stress [27]. Finally, our observations that caloric restriction, reduced weight, HOMA-IR, and triglycerides but increased *Mt-co1* expression and decreased oxidative stress in plaques and in plasma lend further support for MT-COI as a molecular mediator of metabolic dysfunction. We observed that reduced *MT-COI* was related with higher atherosclerotic plaque burden and oxidative stress and was linked with decreases in the Pgc-1α/Ppar pathway reflecting mitochondrial dysfunction [11–14].

A limitation of mouse models is that coronary plaque macrophages cannot be isolated readily. Hence, we measured *MT-COI* in macrophages isolated from coronary atherosclerotic plaques in high-fed diet pigs which are out-bred and develop plaques that are more different in size, composition, and complexity. We used the Stary classification to distinguish the pigs into three groups on basis of the complexity of their plaques. The physiological characteristics of the three groups of pigs were very similar. As only exceptions pigs of the Stary III group had higher HOMA-IR and higher plasma levels of oxidized LDL. Nevertheless, the Stary III group also had lower *MT-COI* and *TFAM* expression in coronary artery plaques, in association with greater plaque size and higher plasma oxidized LDL. Thus, our studies in mice and pigs support the hypothesis that low *MT-COI* is indeed associated with higher oxidative stress related to atherosclerotic plaque size and complexity.

Further important regulators of the COX IV complex comprise the nucleus encoded genes COX4I1 and COX10. The latter is required for COX biogenesis [28]. In turn, COX4I1 is suggested to be the most important regulatory subunit of COX [29,30] as it is required for the allosteric feedback inhibition of the enzyme by its indirect product ATP. Interestingly, *Cox4i1* was also low in aorta of DKO mice, but caloric restriction did not increase Cox4i1. It did also not correlate with oxidative stress and M1 phenotypic switch in macrophages in mice and in bone-marrow-derived macrophages. Expression of *COX4I1* was also lower in macrophages isolated from Stary III than in macrophages isolated from Stary I and Stary II plaques in the LAD of hypercholesterolemic pigs. However, *COX4I1* did not correlate with plaque oxidized LDL. Expression of *COX10* in macrophages was not different according to the plaque type.

A lack of MT-COI knockout prevented us from studying causal relationship between low MT-COI and atherosclerosis. But is unlikely that they can be studied in view of the association of *MT-COI* deficiencies with a wide variety of disorders and the pathological features, consisting of MELAS like syndrome (mitochondrial encephalomyopathy, lactic acidosis, and stroke-like episodes), motor neuron disease, myoglobinuria and sideroblastic anemia in humans [31].

### Regulation of *MT-COI* expression

Our data in preclinical models agree with a role of *TFAM* in the regulation of *MT-COI*. Several lines of evidence indicate that TFAM is required for mtDNA replication and maintenance [32]. Ischemia/Reperfusion injury decreased the protein level of a key activator of mitochondrial transcription, Tfam. [33] In addition, *Tfam* was up-regulated in intimal VSMC of injured rat carotid artery, and suppression of *Tfam* attenuated intimal thickening after balloon injury [34]. Haplogroup *TFAM* gene variation, associated with low *TFAM*, was linked to early-onset myocardial infarction [35]. In our preclinical models, we observed a correlation between *MT-COI* and *TFAM*.

## Conclusions

In conclusion, we found that the expression of low *MT-COI* was associated with high oxidative stress and increased plaque burden in mice and pigs.

## Supporting Information

S1 File Supplementary Methods. (DOCX)
