# Supplementary material for: Low Cytochrome Oxidase 1 Links Mitochondrial Dysfunction to Atherosclerosis in Mice and Pigs
Source: PLoS One. 2017 Jan 25;12(1):e0170307. doi: 10.1371/journal.pone.0170307 (PMC5266248; doi:10.1371/journal.pone.0170307)
Supplement: S1 File — (DOCX) [file pone.0170307.s001.docx]

**S1: supplementary methods**

**Methods**

*Mice*

All mice were sacrificed by Nembutal overdose at the age of 24 weeks [1,2]. After an overnight fast, blood was collected by puncturing the vena cava. Plasma was obtained by centrifugation. Total cholesterol and triglycerides were measured using standard enzymatic colorimetric assays (Boehringer Mannheim, Mannheim, Germany). Glucose was measured with a glucometer (Menarini Diagnostics, Zaventem, Belgium) and plasma insulin with a mouse insulin enzyme-linked immunosorbent assay (Mercodia, Uppsala, Sweden). To determine glucose tolerance, glucose was measured in samples obtained by tail bleeding before and 15, 30, 60, 120 and 240 minutes after intraperitoneal administration of glucose (20% glucose solution; 2 g/kg). Plasma adiponectin and IL-6 were measured with specific mouse ELISA (R&D Systems, Abingdon, UK). The titers of Ig auto-antibodies against oxidized LDL were determined as described previously [3]. The extent of atherosclerosis was determined by analysis of ten 7-µm cross-sections of the aortic valves of DKO mice. Lipids were stained with oil red O and macrophages with an antibody against mouse Mac-3 antigen (Pharmingen, San Diego, CA). Ox-LDL was stained with mAb-4E6. A colour intensity threshold mask for immunoassaying was defined to detect the red colour by sampling, and the same threshold was applied to all specimens. Blinded analysis was performed with the Quantimet 600 image analyzer (Leica, Diegem, Belgium). The positively immunostained area was expressed as a percentage of the total plaque area [4,5].

*Pigs*

Plasma analysis was performed as described for human plasma. Total and HDL-cholesterol and triglyceride levels were determined with enzymatic methods (Boehringer Mannheim). LDL-cholesterol levels were calculated with the Friedewald formula. Plasma glucose was measured with the glucose oxidase method (on Vitros 750XRC, Johnson & Johnson), and insulin with an immunoassay (Biosource Technologies). Adiponectin, oxidized LDL (ox-LDL) and IL-6 were measured with ELISA (Mercodia and R&D Systems). Hs-CRP (Beckman Coulter) was measured on an Image 800 Immunochemistry System; Hs-TnT on a Modular E system (Roche Diagnostics). The extent of coronary atherosclerosis was determined by analysis of 18 cross-sections, spanning a 3-mm segment of the proximal left anterior descending artery (LAD). Macrophages were stained with an anti-MCP1 antibody, ox-LDL with mAb4E6, smooth muscle cells (SMCs) with an anti-α-SM actin monoclonal antibody, and collagen with Sirius red.[6,7] Macrophages (mean: 1000 cells) were microdissected from between 20 and 100 sections spanning a 3-mm proximal segment of the LAD with a PixCell II LCM system using Capture HS LCM caps (Arcturus Engineering, Thermo Fisher, Erembodegem, Belgium). [7] Macrophages were identified on the basis of histological appearance and polyploidy. The adjacent sections were used for plaque phenotyping [8]according to the Stary classification [8,9]. Sections were analyzed using the Leica Quantimet 600 (Leica, Belgium) image processing and analysis system.

**RNA isolation, microarray and quantitative real-time PCR analysis**

Total *RNA* was extracted with TRIzol reagent (Invitrogen, Thermo Fisher, Erembodegem, Belgium) and purified on (mi) RNeasy Mini Kit columns (Qiagen, Vnelo, the Netherlands). *RNA* concentration and quality were assessed with the NanoDrop 2000 (Thermo Scientific, Erembodegem, Belgium), and *RNA* integrity was determined with the RNA 6000 Nano assay kit using the Agilent 2100 Bioanalyzer. First-strand cDNA was generated from total *RNA* with the SuperScript VILO cDNA synthesis kit (Invitrogen). qPCR was performed on a 7500 Fast Real-Time PCR system using Fast SYBRGreen master mix, according to the supplier protocols (Applied Biosystems, Thermo Fisher, Erembodegem, Belgium). Oligonucleotides (Invitrogen) used as forward and reverse mouse and human primers were designed using the “Primer Express” software (Applied Biosystems). Pig primers were designed at the NCBI website. All primers are summarized in S1 table 1. *RNA* expression levels were calculated with the delta-delta-quantification cycle method (ΔΔC_q_) described by Livak and Schmittgen [10]. The C_q_ values for the gene of interest and four stable housekeeping genes were determined for each sample to calculate ΔC_q,sample_ (C_q, gene of interest_ – mean C_q,housekeeping genes_), thus normalizing the data and correcting for differences in amount among *RNA* samples. In detail, *HPRT1*, *SDHA*, *TBP* and *YWHAZ* for pig samples, and *ACTB* for mouse experiments, were selected as most stable housekeeping genes using GeNorm [11]. The expression levels were related to untreated control cells or lean control individuals. Subsequently, ΔΔC_q_ (ΔC_q,sample_ – ΔC_q, control_) was determined, and the relative expression levels were calculated from 2^-ΔΔCq^.

**Supplement table 1: Primers used for qPCR analysis of human RNA extracts**

| **Gene** | **Forward primer** | **Reverse primer** |
| --- | --- | --- |
| **Pig samples** | | |
| *MT-COI* | 5’-CCACGGAAGCAATATGAAATGAT-3’ | 5’-CCTACGTGAAAAGAAAGATGAATC-3’ |
| *COX10* | 5’-GGACTCCCCTCACAAGTTCTTACAT-3’ | 5’-GTGACATACATGCGTTTGAGGAA-3’ |
| *COX4I1* | 5’-GGTCACGCCGATCCATATAAG-3’ | 5’-TCTGTGTGTGTACGAGCTCATGA-3’ |
| *TFAM* | 5’-GCTAGTGGCGGGCATGAT-3’ | 5’-GTGACCCGACCCCAATCTC-3’ |
| *HPRT1* | 5’-CCCTTTCCAAATCCTCAGCAT-3’ | 5’-CCTGGCGTCGTGATTAGTGA-3’ |
| *SDHA* | 5’-CTACCACCACTGCATCAAATTCAT-3’ | 5’-GGAACAAGAGGGCATCTGCTA-3’ |
| *TBP* | 5’-GGAGCTGTGATGTGAAGTTTCCTATA-3’ | 5’-CCAGAAACAAAAATAAGGAGAACAATTC-3’ |
| *YWHAZ* | 5’-TTGATCCCCAATGCTTCACA-3’ | 5’-CGGCAACCTCAGCCAAGT-3’ |

| **Mouse samples** |
| --- |
| \| *Mt-co1* \| *5’-*CCCTAGATGACACATGAGCAAAAG-3’ \| 5’-AGCGTCGTGGTATTCCTGAAA-3’ \| \| --- \| --- \| --- \| \| *Cox10* \| *5’-GGTTGGGTCACAAGCACTCA*-3’ \| 5’-CCGCCCCCAACACACA-3’ \| |
| \| *Cox4i1* \| 5’-CAGCGGTGGCAGAATGTTG-3’ \| 5’-ACACCGAAGTAGAAATGGCTCTCT-3’ \| \| --- \| --- \| --- \| \| *Tfam* \| *5’-*CCCTCGTCTATCAGTCTTGTCTGTAT-3’ \| 5’-ATTTGGGTAGCTGTTCTGTGGAA-3’ \| \| *Cd206* \| 5’-TGTATTCTTTGCCTTTCCCAGTCT-3’ \| 5’-GGATGACAGAGATAAAAGCCAGAAG-3’ \| \| *Ccl2* \| *5’-*GCAGTTAACGCCCCACTCA-3’ \| 5’-CAGCCTACTCATTGGGATCATCTT-3’ \| \| *Pgc-1α* \| 5’-CTGGGTGGATTGAAGTGGTGTAG \| 5’-TATGTTCGCAGGCTCATTGTTG-3’ \| \| *Pparα* \| *5’-*  TCAGGGTACCACTACGGAGTTCA-3’ \| 5’-  CCGAATAGTTCGCCGAAAGA-3’ \| \| *Pparδ* \| *5’-*  CCCCGGTGTAGCCATGAC-3’ \| 5’-  CTCACAGATCACCAGTCCTAAGAACA-3’ \| \| *Ppparγ* \| *5’-*  GCAGCTACTGCATGTGATCAAGA-3’ \| 5’-  GTCAGCGGGTGGGACTTTC-3’ \| |
| \| *β-actin* \| *5’-*ACGGCCAGGTCATCACTATTG-3’ \| 5’-CACAGGATTCCATACCCAAGAAG-3’ \| \| --- \| --- \| --- \| |
|  |
|  |

Abbreviations: MT-COI, cytochrome c oxidase, subunit I; COX, cytochrome c oxidase; HPRT1, hypoxanthine phosphoribosyltransferase 1; Pgc-1*α*, peroxisome proliferator-activated receptor gamma, co-activator 1 alpha; Ppar; peroxisome proliferator-activated receptor; SDHA, succinate dehydrogenase complex, subunit A, flavoprotein (Fp); TBP, TATA box binding protein; TFAM: mitochondrial transcription factoe; YWHAZ, tyrosine 3-monooxygenase/tryptophan 5-monooxygenase activation protein, zeta polypeptide. HPRT1, SDHA, TBP, and YWHAZ were used as housekeeping genes in pig samples; β-actin in mouse samples.

Reference List

1. Mertens A, Verhamme P, Bielicki JK, Phillips MC, Quarck R, Verreth W, et al. . Increased low-density lipoprotein oxidation and impaired high-density lipoprotein antioxidant defense are associated with increased macrophage homing and atherosclerosis in dyslipidemic obese mice: LCAT gene transfer decreases atherosclerosis. Circulation. 2003; 107: 1640-1646.

2. Verreth W, Ganame J, Mertens A, Bernar H, Herregods MC, Holvoet P. Peroxisome proliferator-activated receptor-alpha,gamma-agonist improves insulin sensitivity and prevents loss of left ventricular function in obese dyslipidemic mice. Arterioscler Thromb Vasc Biol. 2006; 26: 922-928.

3. Tsimikas S, Brilakis ES, Miller ER, McConnell JP, Lennon RJ, Kornman KS, et al. Oxidized phospholipids, Lp(a) lipoprotein, and coronary artery disease. N Engl J Med. 353: 46-57.

4. Verreth W, De Keyzer D, Pelat M, Verhamme P, Ganame J, Bielicki JK, et al. Weight-loss-associated induction of peroxisome proliferator-activated receptor-alpha and peroxisome proliferator-activated receptor-gamma correlate with reduced atherosclerosis and improved cardiovascular function in obese insulin-resistant mice. Circulation. 2004; 110: 3259-3269.

5. Geeraert B, Crombé F, Hulsmans M, Benhabiles N, Geuns JM, Holvoet P. Stevioside inhibits atherosclerosis by improving insulin signaling and antioxidant defense in obese insulin-resistant mice. Int J Obes (Lond). 2010; 34: 569-577.

6. De Keyzer D, Karabina SA, Wei W, Geeraert B, Stengel D, Marsillach J, et al. Increased PAFAH and oxidized lipids are associated with inflammation and atherosclerosis in hypercholesterolemic pigs. Arterioscler Thromb Vasc Biol. 2009; 29: 2041-2046.

7. Holvoet P, Davey PC, De Keyzer D, Doukoure M, Deridder E, Bochaton-Piallat ML, et al. Oxidized low-density lipoprotein correlates positively with toll-like receptor 2 and interferon regulatory factor-1 and inversely with superoxide dismutase-1 expression: studies in hypercholesterolemic swine and THP-1 cells. Arterioscler Thromb Vasc Biol. 2006; 26: 1558-1565.

8. Stary HC, Chandler AB, Glagov S, Guyton JR, Insull W, Jr., Rosenfeld ME, et al. A definition of initial, fatty streak, and intermediate lesions of atherosclerosis. A report from the Committee on Vascular Lesions of the Council on Arteriosclerosis, American Heart Association. Circulation. 1994; 89: 2462-2478.

9. Stary HC, Chandler AB, Dinsmore RE, Fuster V, Glagov S, Insull W, Jr., et al. A definition of advanced types of atherosclerotic lesions and a histological classification of atherosclerosis. A report from the Committee on Vascular Lesions of the Council on Arteriosclerosis, American Heart Association. Arterioscler Thromb Vasc Biol. 1995; 15: 1512-1531.

10. Livak KJ, Schmittgen TD. Analysis of relative gene expression data using real-time quantitative PCR and the 2(-Delta Delta C(T)) Method. Methods. 2001; 25: 402-408.

11. Vandesompele J, De Preter K, Pattyn F, Poppe B, Van Roy N, De Paepe A, et al. Accurate normalization of real-time quantitative RT-PCR data by geometric averaging of multiple internal control genes. Genome Biol. 2002; 3: RESEARCH0034.
